# High Schizotypy Predicts Emotion Recognition Independently of Negative Affect

**DOI:** 10.3389/fpsyt.2021.738344

**Published:** 2021-09-23

**Authors:** Christopher Dawes, Claudia Danielmeier, Mark Haselgrove, Paula M. Moran

**Affiliations:** School of Psychology, Nottingham, United Kingdom

**Keywords:** schizotypy, schizophrenia, psychosis, emotion recognition, affect recognition, social cognition, negative affect, mood disorder

## Abstract

**Introduction:** Deficits in Emotion Recognition (ER) contribute significantly to poorer functional outcomes in people with schizophrenia. However, rather than reflecting a core symptom of schizophrenia, reduced ER has been suggested to reflect increased mood disorder co-morbidity and confounds of patient status such as medication. We investigated whether ER deficits are replicable in psychometrically defined schizotypy, and whether this putative association is mediated by increased negative affect.

**Methods:** Two hundred and nine participants between the ages of 18 and 69 (66% female) were recruited from online platforms: 80% held an undergraduate qualification or higher, 44% were current students, and 46% were in current employment. Participants were assessed on psychometric schizotypy using the O-LIFE which maps onto the same symptoms structure (positive, negative, and disorganised) as schizophrenia. Negative affect was assessed using the Depression Anxiety and Stress Scale (DASS-21). Emotion Recognition of both positive and negative emotions was assessed using the short version of the Geneva Emotion Recognition Task (GERT-S).

**Results:** Negative schizotypy traits predicted poorer ER accuracy to negative emotions (β = −0.192, *p* = 0.002) as predicted. Unexpectedly, disorganised schizotypy traits predicted improved performance to negative emotions (β = 0.256, *p* = 0.007) (primarily disgust). Negative affect was found to be unrelated to ER performance of either valence (both *p* > 0.591). No measure predicted ER accuracy of positive emotions. Positive schizotypy traits were not found to predict either positive or negative ER accuracy. However, positive schizotypy predicted increased confidence in decisions and disorganised schizotypy predicted reduced confidence in decisions.

**Discussion:** The replication of ER deficits in non-clinical negative schizotypy suggests that the association between negative symptoms and ER deficits in clinical samples may be independent of confounds of patient status (i.e., anti-psychotic medication). The finding that this association was independent of negative affect further suggests ER deficits in patients may also be independent of mood disorder co-morbidity. This association was not demonstrated for the positive symptom dimension of the O-LIFE, which may be due to low levels of this trait in the current sample.

## Introduction

Schizophrenia is a chronic and potentially debilitating mental-health disorder with an estimated lifetime prevalence of 0.5% (1). The symptoms of schizophrenia can be categorised as positive negative or cognitive (2). The positive symptoms are reality distortion and disorganisation, exemplified by hallucinations, delusions, and disorganised thoughts, speech, and behaviour. Negative symptoms can be broadly categorised as expressive (restricted affect and alogia) and experiential (avolition and apathy, and asociality) (3). Negative symptoms in particular are associated with poorer functional outcomes (4, 5), including lower employment rates (6), smaller social networks (7), and poorer quality of life (8–10). Commonly, it is the negative symptoms and disorganised symptoms that are most consistently associated with poorer social cognition in schizophrenia (11).

Social cognition refers to mental processes responsible for the perception, decoding, interpretation, and regulation of responses to social stimuli (12). In schizophrenia, theory of mind, social perception, attributional bias, and emotion processing have been identified as key domains (13), with deficits reported across the prodromal, first-episode, and multi-episode stages of illness (14–16); suggesting they are an enduring trait marker. Deficits are found relative to both healthy controls (Hedge's *g* = −0.89) (15) and psychiatric controls with bipolar disorder (17), although they are less severe relative to autism spectrum disorders (18). These deficits are thought to underlie inter-personal conflict, isolation, and social disengagement (19) and contribute to poorer functioning. Social cognition may be of particular importance to improving daily functioning as it has been suggested to explain more variance in outcomes than non-social cognition (20–22). Social cognition has also been reported to explain incremental variance over non-social cognition and to mediate the association between non-social cognition (e.g., processing speed, working memory, etc.) and functioning (22). While the presence of social cognitive deficits is well-established in schizophrenia, the mechanisms behind these deficits are not well-understood. This research aims to identify potential explanatory factors in one important domain of social cognition: Emotion Recognition (ER).

In patients, ER performance is negatively associated with reality distortion, negative symptoms, and disorganised symptoms to a similar extent (11). Generally, impairments are found in the perception of negative emotions (sadness and fear) and less consistently in positive emotions, although this may be due to a lack of more varied positive stimuli beyond happiness (12). One potential contributory factor to these deficits is that patients may be hindered by confounds of patient status unrelated to the disease. Antipsychotic medication side-effects (e.g., motor slowness and poor concentration) may artificially inflate cognitive task deficits (23), while social isolation and community exclusion limits opportunities to practise social cognitive skills.

One approach to circumvent these limitations is to assess individuals varying on psychometrically-defined schizotypy; personality traits that reflect the factor structure of symptoms and are a potential marker for the transition to psychosis (24). These schizotypy “symptom” dimensions of positive, negative, and disorganised schizotypy map onto reality distortion, negative symptoms, and disorganised symptoms of schizophrenia, respectively. This dimensional viewpoint considers psychosis a spectrum of behaviour, from non-harmful schizotypy personality traits (e.g., “Do you believe in telepathy?”) to clinical symptoms (e.g., persecutory delusions) that may cause disruption to daily functioning. Some psychometric assessments assess attenuated psychotic-like experiences according to diagnostic criteria (25), such as the Schizotypal Personality Questionnaire (SPQ) which bases its assessment on DSM-III-R criteria for schizotypal personality disorder (26) and the Oxford-Liverpool Index of Feelings and Experiences (O-LIFE) which is partially derived from DSM-II criteria (27). Investigating schizotypy traits allows inferences to be made to behaviour in patients in the absence of clinical confounds (25). Experimentally, if both schizotypy symptom traits in healthy controls and clinical symptoms in patients predict ER performance, this would suggest this relationship is independent of confounds of patient status. Currently, however, findings concerning schizotypy and ER are inconsistent in terms of which dimensions predict performance. Across categorical (“High” vs. “Low” schizotypy) and dimensional (associating traits with performance) approaches, the most consistently implicated traits are negative (28–32) followed by positive (reality distortion) (29, 32, 33), with fewer studies implicating disorganised traits (29, 33). However, other studies have reported no associations for these dimensions: negative (33–38), positive (28, 30, 31, 35, 36, 38–40), and disorganised (28, 30–32, 35, 36). Whether deficits are specific to positive and/or negative emotions is also unclear (28, 31, 41). Moreover, detailed assessments have found no evidence of deficits in disgust (33), but mixed evidence for happiness (36, 42), sadness (33, 36), fear (33, 36), surprise (33, 42), and anger (33, 36, 42). However, deficits have been suggested to be independent of more general face processing deficits (29). Overall, the evidence for ER deficits in schizotypy is currently inconsistent. Consequently, it is unclear whether the schizotypy literature supports the independence of ER deficits in clinical patients from clinical factors such as medication.

One potential reason for this inconsistency may be the confounding role of negative affect. Approximately 23–29% of first episode schizophrenia patients have at least one co-morbid mood disorder (43). For example, Major Depressive Disorder has been associated with poorer recognition of all six basic emotions except sadness (*g* = −0.42 to −0.17) (44). Schizotypy has also been associated with negative affect (45–47). Assessing negative affect in schizotypy may help explain some of the literature inconsistencies. Specifically, if negative affect were to moderate ER deficits in schizotypy, samples high in negative affect would report significant associations while samples low in negative affect may not. Alternatively, both schizotypy and negative affect may contribute to deficits.

Previous research has suggested statistically controlling for negative affect when assessing both schizotypy and ER performance (29, 33). However, only one study to our knowledge has done so. This study found correlations between schizotypy and ER performance remained significant when negative affect was controlled for (28). However, this methodological approach did not allow a comparison of the relative impact of schizotypy and negative affect on ER e.g., by use of a mediation analysis or by comparing standardised effect sizes. Moreover, the tasks used in both this investigation and other previous investigations are limited by the range of positive emotions presented. Commonly, assessments in both schizotypy and schizophrenia use stimuli reflecting Ekman's six basic emotions (48) which includes happiness as a positive emotion. This has been highlighted as a limitation of current research (12) and a potential explanation for inconsistent associations with positive emotions. It is therefore important to include a wider variety of positive emotions (i.e., relief, pleasure, amusement, etc.) which was implemented in the current study.

Consequently, this report aimed to assess whether the literature inconsistencies of ER deficits in schizotypy may be partially explained by increased levels of negative effect. This was investigated by assessing whether negative affect mediates the relationship between schizotypy and ER performance. Normative comparisons of negative affect were also planned to determine whether any lack of mediation may be due to low levels of negative affect in the current sample. If ER deficits are both present in schizotypy and are independent of negative affect, this would suggest deficits in patients are not fully explainable by confounds of patient status (e.g., anti-psychotic medication and social isolation) nor mood disorder co-morbidity, respectively.

Therefore it was hypothesised that: (1) high negative schizotypy (28–32) will predict lower Emotion Recognition accuracy and that (2) if this association is attributable to negative affect it will be reduced if negative affect is controlled for.

## Methods

### Participants

From an initial 232 participants, 23 were excluded (see Data Preparation). The final sample of 209 participants was recruited through the University's recruitment system (15.8%), Call for Participants (15.8%), social media (38.8%), and Prolific (29.7%). In this sample, 66% were biologically female, ages ranged between 18 and 69 years old (*M* = 27.4, *SD* = 10.2), 79.4% had at least an undergraduate level qualification, 44.0% were current students, and 45.9% were currently employed. Of the 148 participants that volunteered responses, 51.6% reported no current medication, 10 participants reported taking anti-depressants, one participant reported taking lithium (a mood stabiliser), but no participant reported anti-psychotic medication. The following analyses did not differ in interpretation when excluding these 11 participants and the remaining participants reported medication such as antihistamines or the contraceptive pill. This study achieved a power of 0.99 for a medium effect size and 0.37 for a small effect size (multiple regression analysis with three predictors) (49).

### Materials

**Figure 1:**
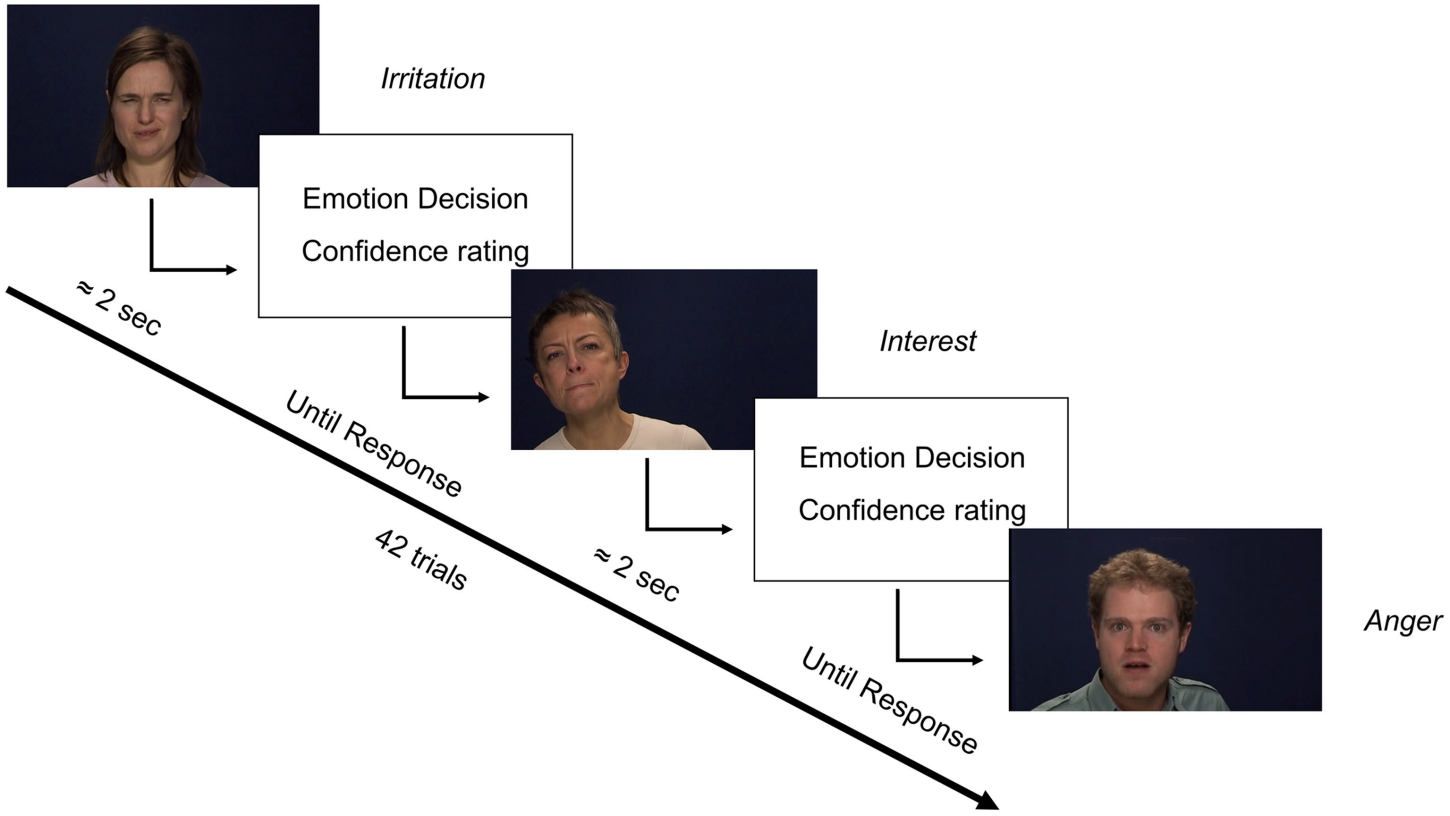
Trial format of the GERT-S with the addition of confidence ratings. Italics represent the emotion displayed by each person. Original materials adapted from (54).

The first three scales of the Oxford-Liverpool Index of Feelings and Experiences (O-LIFE) (50) were used to assess schizotypy. These scales were Unusual experiences (Unex, “Do you believe in telepathy”), Introvertive anhedonia (Intan, e.g., “Do you feel that making new friends isn't worth the energy it takes?), and Cognitive disorganisation (Cogdis, e.g., “Are you easily distracted when you read or talk to someone?”) which correspond to positive (reality distortion), negative, and disorganised schizotypy, respectively. The fourth scale of Impulsive non-conformity was not included as it may not be central to schizotypy (50). Negative affect was assessed using the total score of the Depression, Anxiety, and Stress Scale (DASS-21) (51). A psychometric measure of pre and post-task motivation was also taken using the motivation scale of the Momentary Influences, Attitudes and Motivation Impact (MIAMI) on Cognitive Performance Scale (52). The GERT-S (53), an emotion identification task consisting of 42 items and 14 emotions, was used to assess ER (Figure 1). Stimuli were 1 to 3 s videos of 10 male and female actors. Actors spoke non-sense syllables, meaning recognition was from dynamic facial expression, upper body language, and prosody (but not semantic meaning). Consequently, the task assessed more general emotion recognition. The GERT-S includes high arousal positive (pleasure, relief, interest) and low arousal positive emotions (joy, amusement, pride), and high arousal negative (anger, fear, despair) and low arousal negative items (irritation, sadness, anxiety). Disgust was categorised as negative consistent with most previous reports in schizotypy. Surprised was not categorised as positive nor negative due to conflicting evidence in the wider social cognitive literature. However, as reports on ER in schizotypy primarily consider surprise as positive (28, 32, 41) the analyses were repeated including surprise as a positive emotion, but they did not differ in implication. On each trial participants had to identify which one of the 14 emotions was being presented. To gather more information on decision making an additional scale was added requesting response confidence judgments (from 1 “low confidence” to 7 “high confidence”) that was not present in the original GERT-S. The GERT-S presents good internal consistency (ω_T_ = 0.89) and has been critically reviewed elsewhere (53, 54).

### Procedure

This study was part of a larger cognitive battery also administering two tasks of executive function. All participants complete the study online via Qualtrics between March and August 2020. Psychometric information was collected from one survey while the GERT-S was administered on a separate survey provided by the original authors. The questionnaires were administered first followed by the GERT-S. The GERT-S includes two practise trials, clear definitions of each emotion, and the option to repeat the practise. “Prefer not to say” options were added to all questionnaires for ethical reasons as well as two awareness items which asked participants to select “Prefer not to say.” Psychometric motivation assessments were taken both pre- and post-task (52). Before being debriefed, participants were given the option to withdraw their data if they experienced technical issues or for any other reason (with no justifications required). Participants were compensated with University credits or monetary incentives irrespective of whether they withdrew their data. This study was approved by the University of Nottingham's Ethics Committee (S1214) and all participants gave informed consent.

### Data Preparation

From the original sample, 17 participants were excluded due to failing either awareness item (not selecting “Prefer not to say”), one participant withdrew their data, and one participant was excluded due to excessive “Prefer not to say” responses. Six more were excluded due to outlier performance (total accuracy < median – 2.5 ^*^ Median Absolute Deviation). The exclusion of these participants more readily satisfied model assumptions and but did not affect the current conclusions. Missing data including “prefer not to say” were imputed using the missForest R package (55). Missing data accounted for 0.4, 0.5, and 1.2% of single-item responses for the DASS-21, O-LIFE, and MIAMI respectively.

### Analysis Strategy

The primary outcome was Emotion Recognition accuracy (ER; 0–100%) divided into positive and negative items. To address the first hypothesis each of the O-LIFE scales were added simultaneously to two multiple regressions predicting positive ER and negative ER performance. To assess a potential mediatory role of negative affect, the DASS-21 total score was then added to these multiple regression models. Bayes Factors (BF) were calculated for each regression coefficient to differentiate between data insensitivity and a true null effect (56). BFs were interpreted as follows for the alternate hypothesis (BF_10_): a BF between 3 and 0.333 was insensitive (more data required), BF > 3 moderate evidence, BF > 10 strong evidence, BF > 30 very strong evidence, and BF > 100 decisive evidence (57). As accuracy scores are bound between 0 and 100%, a beta-binomial distribution regression was applied as a robustness cheque. As each approach was identical in conclusions the more readily interpretable Ordinary Least Squares approach is presented. The accuracy of each emotion was correlated with all psychometric scales. Finally, all regression analyses passed the assumptions of normally distributed residuals, linearity, homoscedasticity, lack of influential values (Cook's distance <1), and no multi-collinearity (VIF <5). Analyses were conducted in R studio (58), Jamovi (59), and JASP (60) using several statistical (61, 62) and data visualisation packages (63, 64).

## Results

### Descriptives

Descriptive summaries of psychometric and GERT-S scores can be found in Table 1 and Supplementary Table 1, respectively. The total sample presented lower accuracy score for negative emotions relative to positive emotions [*t*_(208)_ = 7.825, *p* < 0.001, *d* = 0.541]. Normative comparisons were conducted to assess whether current levels of traits were representative of the wider population. As Shapiro-Wilk tests suggested all variables were non-normally distributed (all *p* < 0.011), normative comparisons of the O-LIFE (extracted from the 21 to 30 age category, *N* = 402) (50) and DASS-21 (51) were conducted using One-Sample Wilcoxon signed-rank tests as medians were available. As normative medians are not available for the GERT-S (53) One-Sample *t*-tests were required. Effect sizes for non-parametric tests were rank-biserial correlations (*r*_*rb*_) while *t*-tests used Cohen's *d* (Table 1). Positive schizotypy was lower in the current sample (*p* = 0.004, *r*_*rb*_ = −0.27), negative schizotypy (*p* < 0.001, *r*_*rb*_ = 0.76), DASS-21 (*p* < 0.001, *r*_*rb*_ = 0.80), and total GERT-S were higher (*p* < 0.001, *d* = 1.09), but disorganised schizotypy did not differ (*p* = 0.536, *r*_*rb*_ = 0.04). Scale internal consistency was calculated using McDonald's Omega Total (ω_T_) following recommendations (65). Cronbach's α is presented for completeness but is not appraised due to being unsuitable for psychometric (66) and non-normal data (67). The O-LIFE and DASS-21 presented excellent internal consistency (ω_T_ > 0.80), but the GERT-S was questionable to poor, unlike the original validation. GERT-S scores presented a good range of difficulties and a lack of floor or ceiling effects (Supplementary Table 1). Spearman correlations were also conducted between the three schizotypy scales and both pre and post-task motivation (FDR corrected). The correlations found disorganised schizotypy was significantly associated with lower post-task motivation (*r*_*s*_ = −0.215, *p* = 0.012) and presented a trend association to lower pre-task motivation (*r*_*s*_ = −0.164, *p* = 0.054), but the remaining associations were non-significant (*p* > 0.138).

**Table 1:** Descriptive statistics of psychometric measures compared against normative values.

	**Current**								**Normative**						**Comparison**		
***Scale***	**Range**	**M**	**SD**	**Med**	**MAD**	**IQR**	**ω_T_**	**α**	**Range**	**M**	**SD**	**Med**	**IQR**	**α**	***p***	**ES [Low, High]**	**Norm**
Pos Scz	0–23	8.105	5.587	7.042	5.930	7.967	0.869	0.932	–	10.159	6.304	9^a^	10	0.89	=0.004	−0.27 [−0.16, −0.41]	<0.001
Neg Scz	0–24	8.455	5.594	7.727	5.930	8.425	0.866	0.916	–	5.444	4.000	4.5^a^	6.5	0.82	<0.001	0.76 [0.68, 0.82]	<0.001
Dis Scz	0–24	13.177	6.218	13.600	7.413	9.375	0.899	0.942	–	12.391	5.690	13^a^	–	0.87	=0.536	0.04 [−0.12, 0.20]	<0.001
*Depression*	0–21	7.153	5.610	5.846	5.930	9.117	0.916	0.940	0–21	2.83^b^	3.87	1	–	0.88	<0.001	0.77 [0.62, 0.92]	<0.001
*Anxiety*	0–20	4.746	4.580	3.175	4.448	7.099	0.858	0.900	0–20	1.88^b^	2.95	1	–	0.82	<0.001	0.63 [0.48, 0.77]	<0.001
*Stress*	0–21	7.388	5.154	6.972	5.930	7.982	0.866	0.894	0–21	4.73^b^	4.20	4	–	0.90	<0.001	0.52 [0.37, 0.66]	=0.008
*DASS Total*	0–54	19.287	13.781	15.417	13.343	20.350	0.966	0.958	0–61	9.42^b^	9.66	7	–	0.93	<0.001	0.80 [0.74, 0.85]	=0.011
*GERT-S Tot*	38–83	62.930	10.063	64.172	10.590	12.616	0.535	0.691	–	52^c^	15.318	–	–	0.81−0.83	<0.001	1.09 [0.91, 1.27]	=0.005
*GERT-S Neg*	29–90	60.059	13.748	61.499	14.120	19.294	0.503	0.628	–	–	–	–	–	–	–	–	=0.002
*GERT-S Pos*	39–100	68.979	13.079	71.871	16.473	17.108	0.400	0.562	–	–	–	–	–	–	–	–	=0.001

*IQR, interquartile range; MAD, median absolute deviation, medians are interpolated medians; **ω_T_**, McDonald's Omega total; Norm, Shapiro-Wilk tests. Group comparisons are One-Sample Wilcoxon Sign-Rank tests for both the O-LIFE and DASS-21 and a One Sample t-tests for the GERT-S (all p-values are False Discovery Rate corrected), ES = rank-biserial correlations for Wilcoxon and Cohen's d for t-tests*.

a *Mason and Claridge (50)*,

b *Henry and Crawford (51)*,

c *Schlegel and Scherer (53)*.

### Emotion Recognition Accuracy

Two multiple regression analyses (Table 2) entered the three O-LIFE scales as predictors of positive and negative accuracy. For negative emotions, negative schizotypy predicted poorer performance (β = −0.192[−0.333, −0.052], *p* = 0.007, BF_10_ = 3.238, *R*^2^_partial_ = 3.5%), disorganised schizotypy predicted improved performance at a larger effect size (β = 0.256[0.096, 0.417], *p* = 0.002, BF_10_ = 4.387, *R*^2^_partial_ = 4.6%), but positive schizotypy returned non-significant with the BF_10_ suggesting more data were needed to accept the null hypothesis (*p* = 0.094, BF_10_ = 0.671) (hypothesis 1). All significant associations survived FDR correction for multiple comparisons (all *p* < 0.021). For positive emotions, no O-LIFE scale significantly predicted performance (all *p* > 0.090). The BFs suggested there was moderate evidence for null hypothesis for positive schizotypy (BF_10_ = 0.191) and disorganised schizotypy (BF_10_ = 0.256), but more data were needed to conclude about negative schizotypy (BF_10_ = 0.477). Both sex and age were not significant predictors of performance when added to these two regression models and did not affect the significant associations between schizotypy and performance.

**Table 2:** Multiple linear regressions predicting positive and negative emotion recognition accuracy from positive (Unex), negative (Intan), and disorganised (Cogdis) schizotypy.

	**Pos Accuracy**								**95% Conf Int**	
**Predictor**	**B**	**SE**	**t**	***p***	**BF_**10**_**	$R_{partial}^{2}$	**VIF**	**β**	**LC**	**HC**
Intercept	69.480	2.337	29.727	<0.001						
Pos Scz	−0.106	0.187	−0.565	=0.572	0.191	1.156	1.326	−0.045	−0.202	0.112
Neg Scz	−0.290	0.170	−1.706	=0.090	0.477	1.399	1.105	−0.124	−0.268	0.019
Dis Scz	0.213	0.175	1.217	=0.225	0.256	0.718	1.447	0.101	−0.063	0.266
	**Neg Accuracy**								**95% Conf Int**	
**Predictor**	**B**	**SE**	**t**	***p***	**BF**_**10**_	$R_{partial}^{2}$	**VIF**	**β**	**LC**	**HC**
Intercept	59.202	2.400	24.666	<0.001						
Pos Scz	−0.323	0.192	−1.683	=0.094	0.671	1.362	1.326	−0.131	−0.285	0.023
Neg Scz	−0.473	0.175	−2.706	=0.007	3.238	3.449	1.105	−0.192	−0.333	−0.052
Dis Scz	0.567	0.180	3.152	=0.002	4.387	4.622	1.447	0.256	0.096	0.417

***Positive:** F_(3, 205)_ = 1.152, p = 0.329, R^2^ = 1.7%, $R_{adjusted}^{2}$ = 0.2%. **Negative:** F_(3, 205)_ = 4.469, p =0.005, R^2^ = 6.1%, $R_{adjusted}^{2}$ = 4.8%. VIF, Variance Inflation Factor. Bayesian priors are full Cauchy (location = 0, scale = 0.354)*.

**Figure 2:**
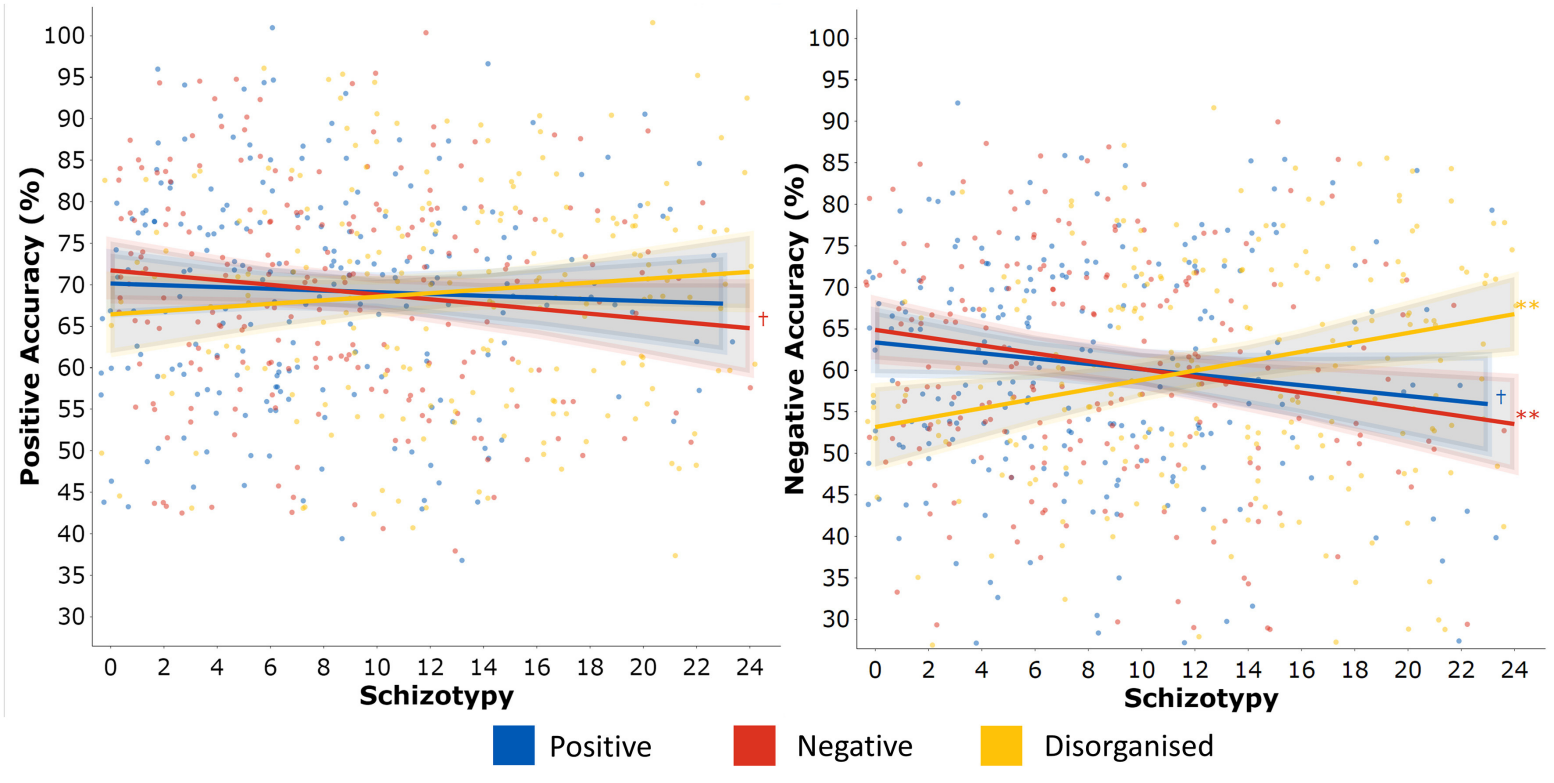
Post estimation of multiple linear regressions predicting positive (left) and negative (right) emotion recognition accuracy from positive (Unex), negative (Intan), and disorganised (Cogdis) schizotypy.

Total DASS-21 score did not predict positive (β = −0.037 [−0.174, 0.100], *p* = 0.591, BF_10_ = 0.172) nor negative ER accuracy (β = 0.014[−0.123, 0.151], *p* = 0.843, BF_10 =_ 0.153). Unplanned exploratory analyses additionally confirmed no DASS-21 subscale predicted performance on the GERT-S that would warrant more detailed investigation (positive ER: Depression, *p* = 0.536, Anxiety, *p* = 0.333, Stress, *p* = 0.922; negative ER: Depression, *p* = 0.867, Anxiety, *p* = 0.890, Stress, *p* = 0.638). The inclusion of DASS-21 total score these regression models in Table 2. did not change the associations between schizotypy and both positive and negative ER accuracy. Consequently, this suggested negative affect does not mediate the relationship between schizotypy and ER performance (hypothesis 2). Marginal effects were plotted to illustrate the independent effects of each O-LIFE scale on negative ER accuracy (Figure 2). Participants scoring in the 90th percentile of positive schizotypy, negative schizotypy, or disorganised schizotypy were predicted to have changes in accuracy of −4.52%, −7.1%, and +9.08% respectively, relative to participants scoring in the 10th percentile (a common cut-off criterion for categorical studies).

### Individual Emotion Recognition Accuracy

A spearman's correlation matrix was calculated between the O-LIFE, DASS-21, and emotion recognition accuracy (Table 3). The effect of negative schizotypy for negative items may have come from anger and fear (both *p* < 0.074), although these analyses were both trend and did not survive FDR correct (both *p* < 0.395). The effect of disorganised schizotypy for negative items likely came from disgust (*r*_*s*_ = 0.254, *p* < 0.001, *p*_*FDR*_ = 0.007). To assess the latter, the multiple regression analysis was repeated with the exclusion of disgust. Disorganised schizotypy remained a significant albeit weaker predictor (β = 0.196 [0.034, 0.357], *p* = 0.018). A conflicting negative correlation between disorganised schizotypy and relief accuracy was also found (*r*_*s*_= −0.139, *p* = 0.045), but this did not survive FDR correction (*p* =0.277). This association may be an indirect result of the association between disorganised schizotypy and DASS-21 total score (*r*_*s*_ = 0.681, *p*_*FDR*_ = 0.007), as the DASS-21 was also correlated with relief accuracy in the same direction (*r*_*s*_ = −0.186, *p*_*FDR*_ = 0.050). In keeping with the regression results, positive schizotypy was not correlated with any emotion. Correlations between psychometric scales and reaction time are provided in Supplementary Table 2.

**Table 3:** Spearman correlations between the accuracy of each emotion and schizotypy.

			**Schizotypy**				**Negative Affect**
**Valance**	**Arousal**	**Scale**	**Total**	**Pos**	**Neg**	**Dis**	**DASS Total**
		Pos	0.718^***^	–			
		Neg	0.608^***^	0.122	–		
		Dis	0.834^***^	0.490^***^	0.300^***^	–	
		DASS Total	0.634^***^	0.404^***^	0.309^***^	0.681^***^	–
**Positive**	High	Interest	−0.100	−0.106	−0.091	−0.071	−0.065
		Pleasure	0.074	0.046	0.065	0.049	0.012
		Relief	−0.113	−0.047	−0.038	−0.139^*^	−0.186^**^
	Low	Amusement	0.034	0.035	−0.079	0.097	0.069
		Joy	−0.040	−0.011	−0.105	0.036	0.059
		Pride	0.035	−0.016	0.002	0.061	−0.036
**Negative**	High	Anger	−0.018	0.058	−0.124^†^	0.050	0.054
		Fear	−0.024	−0.009	−0.131^†^	0.069	0.067
		Despair	0.009	0.000	−0.024	0.020	0.047
	Low	Anxiety	0.021	0.024	0.027	0.025	−0.038
		Irritation	0.003	−0.076	0.001	0.083	0.011
		Sadness	−0.083	−0.001	−0.109	−0.050	−0.016
	NR	Disgust^a^	0.167^*^	0.072	−0.012	0.254^***^	0.068
	NR	Surprise	−0.030	0.023	−0.037	−0.054	−0.013

a *Schlegel and Scherer (53) did not suggest arousal of disgust*,

† *p < 0.1,*

* *p < 0.05,*

** *p < 0.01,*

*** *p < 0.001*.

*Unex, Unusual Experiences; Intan, Introvertive Anhedonia; Cogdis, Cognitive Disorganisation; DASS Total, Depression, Anxiety, and Stress Scale total score. Significant correlations between Intan and both anger (p = 0.395) and fear (p = 0.337) recognition and Cogdis and Relief accuracy (p = 0.277) do not survive correction for multiple comparison (False Discovery Rate, FDR). However, the associations between Cogdis and disgust (p = 0.007) and DASS total score and Relief accuracy (p = 0.050) do survive correction*.

### Response Confidence

Regression analyses were repeated with GERT-S decision confidence as the outcome. Originally, four multiple regressions were conducted dividing responses between both valence (positive vs. negative) and veracity (correct vs. incorrect decisions). However, as the relationship between schizotypy and confidence rating were unaffected by these variables and splitting the analyses violated several model assumptions, only overall confidence is presented. A multiple regression analysis (Table 4) reported that positive schizotypy marginally predicted greater confidence, but the BF_10_ suggested more data were needed (β = 0.166 [0.012, 0.320], *p* = 0.035, BF_10_ = 1.358) and the association became trend under FDR correction (*p* = 0.053). In contrast, disorganised schizotypy predicted reduced confidence (β = −0.220 [−0.381, −0.059], *p* = 0.008, *p*_FDR_ = 0.024, BF_10_ = 3.892). More data were needed to conclude about negative schizotypy which returned non-significant (*p*_*FDR*_ =0.453, BF_10_ = 0.497). Total DASS-21 was not found to predict confidence judgements, but the BF_10_ suggested more data were needed (β = −0.107 [−0.244, 0.029], *p* = 0.122, BF_10_ = 0.465).

**Table 4:** Multiple linear regressions predicting response confidence to either correct or incorrect decisions from positive (Unex), negative (Intan), and disorganised (Cogdis) schizotypy dimensions.

	**Confidence**								**95% Conf Int**	
**predictor**	**β**	**SE**	**t**	***p***	**BF_**10**_**	** $R_{partial}^{2}$ **	**VIF**	**β**	**LC**	**HC**
Intercept	5.190	0.147	35.385	<0.001						
Pos Scz	0.025	0.012	2.122	=0.035	1.358	2.150	1.326	0.166	0.012	0.320
Neg Scz	−0.011	0.011	−1.061	=0.290	0.497	0.546	1.105	−0.076	−0.217	0.065
Dis Scz	−0.030	0.011	−2.695	=0.008	3.892	3.421	1.446	−0.220	−0.381	−0.059

*F_(3, 205)_ = 3.910, p = 0.010, R^2^ = 5.4%, $R_{adjusted}^{2}$ = 4.00%. VIF, Variance Inflation Factor. Bayesian priors are full Cauchy (location = 0, scale = 0.354)*.

## Discussion

This study assessed Emotion Recognition (ER) in psychometrically-defined schizotypy (measured using the O-LIFE). The first hypothesis that negative schizotypy would predict poorer ER performance was supported. The second hypothesis that negative affect (measured using the DASS-21 total score) would mediate deficits in schizotypy was not supported. Unexpectedly, we found that disorganised schizotypy predicted improved performance and whether positive schizotypy was related to performance was inconclusive. The effects of negative schizotypy and disorganised schizotypy were statistically significant for negative but not positive emotions. The standardised effect sizes of negative and disorganised schizotypy here (β = −0.192 to 0.256) were much larger than one previous study using a similar approach (*N* = 2,332, β = −0.04 to −0.10) (29). Positive schizotypy marginally predicted higher confidence in decisions, while disorganised schizotypy predicted reduced confidence in decisions.

The finding that schizotypy was associated with performance on negative emotions is consistent with reviews using a variety of emotion recognition instruments in patients with schizophrenia (12). This replication may suggest ER is a valid construct to investigate the dimensional aspects of schizophrenia in the absence of clinical confounds. As the schizotypy literature is equivocal, these findings confirm some but not all studies (28, 31, 33, 34, 36, 38, 41, 42). One reason why only negative emotions may have shown differential effects is that they may activate unpleasant internal states in participants; producing excessive anxiety that can be detrimental to performance. Although negative affect was unrelated to performance, the DASS-21 is not suitable to determine this as it assesses trait rather than state disturbances. To test this hypothesis, state anxiety questionnaires or physiological measures (e.g., Galvanic Skin Response) could be applied. Alternatively, perhaps the GERT-S itself is not sensitive to detect deficits, as the use of multi-modal stimuli (prosody, body language, facial expression) may provide adequate information for processing. This may be consistent with only the more difficult negative emotions being predicted by schizotypy and not the less difficult positive emotions. The lack of significant association between positive emotion recognition and schizotypy is also consistent with some (31, 38, 41) but not all past investigations (28, 32, 34, 42). As there are currently no investigations in schizotypy or schizophrenia that compares performance to controls on the GERT-S, it cannot be ruled out that our findings are due to the ER instrument used. However, one study has assessed patients using the GERT-S (with no control group) and reported an average score of 53.5% (68). The average score in this control sample was 62.9% which may suggest the GERT-S is sensitive to detect ER deficits in psychosis patients. However, non-clinical participants in the original validation of the GERT-S (averaged across both studies) scored 52%, thus indirect comparisons in this case may not insightful. Consequently, future research should aim to replicate these ER deficits in clinical patients relative to a control group. Due to the employment of the GERT-S, however, a lack of diverse positive stimuli is an unlikely explanation for our findings, which is a commonly cited limitation of previous emotion recognition research (12).

The explanations above are likely only applicable to negative schizotypy, which predicted poorer ER performance consistent with previous research in patients (11) and adds to equivocal research in schizotypy (28–32). One clinical study reported 20% of the variance in ER performance was explained by negative symptoms (69). The effect of negative schizotypy in our non-clinical sample was lower (3.5%) which was expected given the dimensional view of psychosis as a spectrum (i.e., less severe deficits should occur with less severe schizophrenia-like experiences). The correlational analysis suggested the deficits in ER were potentially due to poorer fear and anger recognition (but these correlations did not survive correction for multiple comparisons). These potential associations are, however, consistent with previous findings in patients (12). Previous research has found items on social anxiety may primarily drive the effect in negative schizotypy (30). It has been suggested that poor ER may increase social anxiety through reduced confidence in social cognitive abilities (29), perhaps leading to increased social withdrawal and negative traits (35). However, in this study, negative schizotypy did not predict confidence in decisions which conflicts with this suggestion.

Another explanation could be that this relationship is mediated through increased alexithymia, which is increased in clinical samples (70) and correlates with all three schizotypy trait dimensions (71, 72). This initially contradicts the current explanation being specific to negative schizotypy. However, without controlling for scale inter-correlation, it is unclear whether these associations are general or scale specific. If this suggestion were accurate, the experiential rather than expressive negative traits would correlate with self-reported alexithymia. To the best of our knowledge, no study has controlled for alexithymia in this context. One study has assessed alexithymia, but because performance was unaffected by schizotypy, further investigation was unnecessary (39).

The replication of ER deficits in negative schizotypy may suggest the association between negative symptoms and poorer ER in clinical samples may not an artefact of patient status. Moreover, as these deficits were found to be independent of negative affect this may also suggest that mood disorder co-morbidity may not completely explain ER deficits in schizophrenia patients. However, it should be stated that the internal consistency for both positive and negative emotion was low (Table 1), which should caution interpretation.

This study is the first to report a positive association between disorganised schizotypy and ER. This conflicts with previous research in schizotypy commonly reporting no associations (28–31, 33, 35, 36) and patient samples finding negative associations (11). The improved performance in this study was driven primarily through disgust recognition, which contradicts impaired disgust recognition in patient samples (12) and schizotypy samples (29, 33). Both increased deliberation time and improved motivation are unlikely explanations for this improved performance as all schizotypy dimensions generally correlated with increased reaction time (Supplementary Table 2) and disorganised schizotypy correlated with reduced post-task motivation. Studies have reported that schizotypy can exaggerate the perceived emotion expressed in ER tasks (73), which may lead to improved ER performance. However, performance benefits are commonly found in the paranoid subtype of patients (74) and paranoia-related (positive) schizotypy (73), rather than disorganised schizotypy. Alternatively, perhaps participants that can more accurately identify negative emotions have a negatively biassed perception of social interactions. This psychological stress may in turn lead to reports of disorganised thinking. Finally, as this is the only study in schizotypy to use the O-LIFE (rather than the SPQ) or the GERT-S, these results may be specific to the conceptualisations of these measures.

Previously, it has been suggested that positive schizotypy traits such as paranoia may bias participants to expect negative facial emotions and response, or that poorer ER may make individual highly suspicious (32). This is consistent with ER deficits correlating with positive symptoms in patient samples (11) but contrasts with the majority of non-clinical samples (28, 30, 31, 35, 36, 38–40). However, the Bayesian analyses in the current study suggested more evidence was needed to support a lack of relationship. The disparity between clinical and non-clinical studies may be explained by very high levels of positive schizotypy traits being necessary to produce deficits. Indeed, negative schizotypy has been reported to only correlate with FER performance in those classified as being high in schizotypy (31). In this study the normative comparisons found that the levels of positive schizotypy were significantly lower in the current sample, but negative schizotypy and disorganised schizotypy were not, which both predicted performance.

Positive schizotypy predicted increased confidence in decisions while disorganised schizotypy predicted decreased confidence. Clinical studies using both social and non-social stimuli have suggested patients are underconfident in correct responses and overconfident in errors (75); which may underlie impaired functioning and delusion formation, respectively. However, confidence here was unaffected by both valance and veracity, suggesting a divergence with past research in patient samples (75). The discrepancy between patient and schizotypy samples may highlight a potential cognitive mechanism subject to deterioration at illness onset. As a clinical diagnosis is often the result of positive symptoms and is associated with a decline in social cognition, this overconfidence would likely be applied to now impaired performance. This overconfidence in positive schizotypy may be highly relevant to delusion formation. However, it is important to state that the BF_10_ suggested only anecdotal evidence of this association which should caution interpretations. The under-confidence associated with disorganised schizotypy may potentially explain the beneficial effects of this trait in the current study. Although deliberation time and motivation were unlikely explanations, under-confidence may produce more effortful deliberation. This would suggest disorganised schizotypy may relate to a more general cognitive thinking style that is independent of judgement veracity. When combined with the results on accuracy, this suggests (a) positive schizotypy predicts ER overconfidence but intact accuracy, (b) disorganised schizotypy predicts ER underconfidence but improved accuracy, and (c) negative schizotypy predicts poorer ER accuracy with unaffected confidence judgments. If the beneficial effect of disorganised schizotypy can be replicated, improving decision confidence for those high in disorganised schizotypy may improve the transferal of skills to real-world functioning, which may also be relevant to patient samples. This finding of alternated metacognitive processing in schizotypy merits further investigation, potentially with the addition of psychometric metacognitive scales.

Several limitations of this study should be highlighted. Firstly, while the advantage of using a schizotypy sample is the removal of clinical confounds, caution must be applied when applying conclusions directly to clinical samples. Secondly, the cross-sectional nature of this study means that the results are associative and causality cannot be determined. Thirdly, we used single assessments of ER, schizotypy and negative affect which may limit this pattern of results to the specific instruments used.

## Conclusions

This study demonstrated Emotion Recognition deficits were associated with negative schizotypy, suggesting an association between negative clinical symptoms and emotion recognition deficits may be independent of confounds of patient status (i.e., anti-psychotic medication). Inconclusive evidence was found for an association with positive schizotypy (BF_10_ = 0.671), which may be explained by the low levels of positive schizotypy traits in the current investigation. Unexpectedly, disorganised schizotypy predicted improved recognition which may be due to under-confidence in decisions increasing effortful deliberation. Negative affect was found to not mediate reduced Emotion Recognition performance; potentially suggesting that impairments in clinical patients may be independent of co-morbid mood disorders. This has implications for therapeutic interventions and merits further investigation in a clinical sample.

## Data Availability Statement

The raw data supporting the conclusions of this article will be made available by the authors, without undue reservation.

## Ethics Statement

The studies involving human participants were reviewed and approved by University of Nottingham Ethics Committee (S1214). The patients/participants provided their written informed consent to participate in this study.

## Author Contributions

CDaw designed the study, conducted the literature review and statistical analyses, and wrote the first draft. PM designed the study and reviewed both the first draft and final manuscript. MH and CDan reviewed early conceptualisations of the project and contributed to academic discussion and reviewed several draughts of the manuscript. All authors have reviewed and approved the final manuscript.

## Funding

CDaw was funded by a University of Nottingham Economic and Social Research Council Doctoral Training Centre grant [ES/J500100/1] as part of their Ph.D. All other authors report their contributions were supported by no specific grant from any funding agency, commercial or not-for-profit sectors.

## Conflict of Interest

The authors declare that the research was conducted in the absence of any commercial or financial relationships that could be construed as a potential conflict of interest.

## Publisher's Note

All claims expressed in this article are solely those of the authors and do not necessarily represent those of their affiliated organizations, or those of the publisher, the editors and the reviewers. Any product that may be evaluated in this article, or claim that may be made by its manufacturer, is not guaranteed or endorsed by the publisher.

## Abbreviations

ER: Emotion Recognition.
