# Supplementary material for: High Schizotypy Predicts Emotion Recognition Independently of Negative Affect
Source: Front Psychiatry. 2021 Sep 23;12:738344. doi: 10.3389/fpsyt.2021.738344 (PMC8495418; doi:10.3389/fpsyt.2021.738344)
Supplement: Supplementary file 1 [file Data_Sheet_1.docx]

Supplementary Material

**Supplementary Table 1.** Descriptive statistics of the GERT-S items split by emotion.

| **Emotion** |  | **Items Correct (%)** | | | |  | **Confidence (1 – 7)** | | | | |  |
| --- | --- | --- | --- | --- | --- | --- | --- | --- | --- | --- | --- | --- |
|  |  | 0 | 1 | 2 | 3 |  | *M* | *SD* | Median | MAD | IQR |  |
| *Interest* |  | 0.5 | 10.5 | 40.2 | 48.8 |  | 4.7 | 1.17 | 4.81 | 0.99 | 1.33 |  |
| *Amusement* |  | 4.8 | 10.5 | 34.0 | 50.7 |  | 5.6 | 1.04 | 5.89 | 0.99 | 1.33 |  |
| *Anger* |  | 1.4 | 13.9 | 39.7 | 45.0 |  | 5.6 | 1.05 | 5.94 | 0.99 | 1.33 |  |
| *Pleasure* |  | 2.9 | 14.4 | 34.4 | 48.3 |  | 5.32 | 1.07 | 5.42 | 0.99 | 1.67 |  |
| *Relief* |  | 2.4 | 16.3 | 40.7 | 40.7 |  | 5.01 | 1.13 | 5.1 | 0.99 | 1.33 |  |
| *Sadness* |  | 4.8 | 21.5 | 38.8 | 34.9 |  | 4.89 | 1.13 | 4.89 | 0.99 | 1.67 |  |
| *Joy* |  | 6.7 | 24.4 | 34.4 | 34.4 |  | 5.31 | 1.08 | 5.49 | 0.99 | 1.33 |  |
| *Irritation* |  | 6.7 | 23.0 | 37.3 | 33.0 |  | 4.45 | 1.19 | 4.25 | 1.48 | 1.67 |  |
| *Disgust* |  | 4.8 | 27.8 | 50.2 | 17.2 |  | 4.7 | 1.07 | 4.6 | 0.99 | 1.33 |  |
| *Despair* |  | 8.6 | 26.8 | 40.7 | 23.9 |  | 5.06 | 1.06 | 5.12 | 0.99 | 1.33 |  |
| *Fear* |  | 15.8 | 30.6 | 34.0 | 19.6 |  | 4.74 | 1.12 | 4.78 | 0.99 | 1.67 |  |
| *Surprise* |  | 9.6 | 46.9 | 37.3 | 6.2 |  | 4.35 | 1.22 | 4.66 | 1.48 | 2 |  |
| *Pride* |  | 18.2 | 43.1 | 30.1 | 8.6 |  | 4.61 | 1.07 | 4.6 | 0.99 | 1.33 |  |
| *Anxiety* |  | 25.8 | 37.3 | 32.5 | 4.3 |  | 4.35 | 1.09 | 4.38 | 0.99 | 1.33 |  |
| **Note:** IQR – interquartile range, MAD = Median Absolute Deviation, medians are interpolated medians (nearest integer is true median). | | | | | | | | | | | | |

| **Supplementary Table 2.** Spearman correlations between reaction time of each emotion and schizotypy | | | | | | | | |
| --- | --- | --- | --- | --- | --- | --- | --- | --- |
|  |  |  | **Schizotypy** | | | |  | **Negative Affect** |
| **Valance** | **Arousal** | **Scale** | **Total** | **Pos** | **Neg** | **Dis** |  | **DASS  Total** |
|  |  | Pos | 0.718^***^ | - |  |  |  |  |
|  |  | Neg | 0.608^***^ | 0.122^†^ | - |  |  |  |
|  |  | Dis | 0.834^***^ | 0.490^***^ | 0.300^***^ | - |  |  |
|  |  | DASS Total | 0.634^***^ | 0.404^***^ | 0.309^***^ | 0.681^***^ |  | - |
| **Positive** | High | Interest | 0.132^†^ | 0.075 | 0.070 | 0.139^*^ |  | 0.076 |
|  |  | Pleasure | 0.083 | 0.069 | 0.058 | 0.078 |  | 0.055 |
|  |  | Relief | 0.099 | 0.079 | 0.020 | 0.104 |  | 0.128 |
|  | Low | Amusement | 0.158^*^ | 0.131^†^ | 0.101 | 0.135^†^ |  | 0.103 |
|  |  | Joy | 0.115 | 0.015 | 0.124^†^ | 0.126^†^ |  | 0.052 |
|  |  | Pride | -0.005 | -0.065 | 0.045 | 0.031 |  | -0.013 |
| **Negative** | High | Anger | 0.071 | 0.015 | 0.057 | 0.051 |  | 0.055 |
|  |  | Fear | 0.149^*^ | 0.042 | 0.180^**^ | 0.124^†^ |  | 0.032 |
|  |  | Despair | 0.083 | 0.094 | 0.068 | 0.059 |  | 0.081 |
|  | Low | Anxiety | 0.061 | 0.012 | 0.100 | 0.049 |  | -0.042 |
|  |  | Irritation | 0.042 | 0.031 | 0.012 | 0.018 |  | -0.015 |
|  |  | Sadness | 0.137^*^ | 0.101 | 0.091 | 0.118^†^ |  | 0.169^*^ |
|  | NR | Disgust^a^ | 0.173^*^ | 0.109 | 0.112 | 0.163^*^ |  | 0.175^*^ |
|  | NR | Surprise | -0.005 | -0.001 | 0.026 | -0.039 |  | -0.077 |
| **Note**: ^a^ = Schlegel & Scherer (2016) did not suggest arousal of disgust, ^†^= *p* < .1, ^*^ = *p* < .05, ^**^ = *p* < .01, ^***^ = *p* < .001. Unex = Unusual Experiences, Intan = Introvertive Anhedonia, Cogdis = Cognitive Disorganisation, DASS Total = Depression, Anxiety, and Stress Scale total score. | | | | | | | | |
